# Reply to a letter from Miesbach and Srivastava: response to hemophilia B gene therapy based on adeno-associated virus serotype 5 antibody status

**DOI:** 10.1016/j.rpth.2026.103448

**Published:** 2026-03-26

**Authors:** Robert Klamroth, Paul E. Monahan, Sandra Le Quellec

**Affiliations:** 1 Internal Medicine, Vascular Medicine and Coagulation Disorders, Vivantes Clinic Friedrichshain, Berlin, Germany; 2 Gene Therapy, Chapel Hill, North Carolina, USA; 3 CSL Behring, King of Prussia, Pennsylvania, USA

Dear Editors,

We appreciate Professors Miesbach and Srivastava’s analysis of data from 2 recent post hoc subgroup analyses from the HOPE-B trial of etranacogene dezaparvovec gene therapy for hemophilia B, published in *Research and Practice in Thrombosis and Haemostasis* [1,2]. These studies reported sustained clinical outcomes in individuals with or without preexisting adeno-associated virus serotype 5 (AAV5) neutralizing antibodies (NAbs) at the time of infusion. While prior HOPE-B publications presented aggregate outcomes for the entire trial population [3,4], our intention in providing 48-month follow-up data segregated by these subgroups is to facilitate shared decision-making by clinicians and persons with hemophilia when considering screening for AAV5 NAb status prior to treatment. The careful synthesis of these 2 reports provided by Miesbach and Srivastava offers valuable perspectives; in this letter, we provide additional insights.

Miesbach and Srivastava highlight that detectable AAV NAbs at baseline have traditionally been an exclusion criterion for systemically administered AAV-based vector therapies. This paradigm was first challenged in the etranacogene dezaparvovec phase 2b trial (*n* = 3) [5] and abandoned during enrollment in HOPE-B (*n* = 54). Although AAV5 NAb status was documented at enrollment, prior to treatment, and throughout the trial, all aspects of treatment and outcome documentation were identical in the subgroups of participants with and without AAV5 NAb.

Miesbach and Srivastava conclude that efficacy and overall safety outcomes were similar between the NAb^−^ and NAb^+^ subgroups but note a higher incidence of infusion-related reactions (IRRs) in the NAb^+^ subgroup, specifically at higher AAV5 NAb titers. The observed IRRs were infrequent and generally manageable, highlighting the feasibility and importance of administering the full gene therapy dose. Of the 2 of 57 participants treated in phase 2b and HOPE-B, who experienced a lack of efficacy and failed to discontinue factor IX concentrate prophylaxis, 1 was the participant whose infusion was terminated after receiving only ∼10% of the dose. To aid consideration of peri-infusional management of etranacogene dezaparvovec, the Table provides individual data for the 7 participants (5 NAb^+^ and 2 NAb^−^) who reported an adverse event coinciding with the infusion of gene therapy.

**Table:** IRR from phase 2b and HOPE-B trials.

Patient	Preexisting AAV5 NAb status: negative/positive (titer)	AEs verbatim	Severity of AEs	Action taken with the drug (% dose administered)	Additional medication/therapy	FIX activity levels at month 6 postgene therapy
1^a^	Positive (199)	Suspected hypersensitivity reaction	Moderate	Drug infusion withdrawn (∼10%)	Diphenhydramine i.v., methylprednisolone i.v., famotidine i.v., epinephrine i.m., Demerol i.v., and NS bolus (0.9%) i.v.	NA^b^
2	Positive (558)	Infusion site reaction, right eye itchiness, hives behind right ear, headache, and dizziness	Mild	Drug infusion paused, restarted, and completed (100%)	Diphenhydramine p.o. and i.v.; hydrocortisone i.v.	28.4
3	Negative (<LOD^c^)	Dizziness, chest tightness, and flushing	Moderate	Drug infusion paused, restarted, and completed (100%)	Diphenhydramine i.v.	15.9
4	Negative (<LOD^c^)	Fever	Mild	Drug infusion completed with no pause and no change of infusion rate (100%)	—	62.4
5	Positive (482)	IRR: facial flushing, feeling cold, shivers, and rise in blood pressure	Mild	Drug infusion paused, restarted at slower rate (ie, 250 mL/h), and completed (100%)	Chlorphenamine i.v. and hydrocortisone i.v.	29.2
6^a^	Positive (3212)	Epigastric pain	Mild	Drug infusion completed with no pause and no change of infusion rate (100%)	—	NA^b^
7	Positive (23)	IRR, itching, tightness of throat, and swelling neck	Mild	Drug infusion completed with no pause and no change of infusion rate (100%)	—	37.9

AAV5, adeno-associated virus serotype 5; AE, adverse event; FIX, factor IX; i.v., intravenous; i.m., intramuscular; IRR, infusion-related reaction; LOD, limit of detection; NA, not applicable; NAb, neutralizing antibody; NS, normal saline; p.o, per oral.

a See details in the extended narrative for this participant, published in the Supplemental Materials of [6].

b Participants did not have evaluable uncontaminated endogenous FIX activity levels at month 6 postgene therapy as they remained on continuous FIX prophylaxis following administration of etranacogene dezaparvovec.

c LOD = 7.

Miesbach and Srivastava note an apparent NAb titer–dependent IRR association. Reviewing all NAb^−^ and NAb^+^ participants in phase 2b and HOPE-B, 51 participants had preexisting NAb titers ranging from undetectable to a titer of 115, associated with 3 reports of infusion-related symptoms (3/51, 5.9%); 2 of these participants received no intervention or change in the infusion. In contrast, 6 participants had preexisting NAb of ≥198.9 associated with 4 reports of infusional symptoms (4/6, 66.7%), and 1 of these 4 episodes received no intervention. No participants received a dose of premedication (eg, with single-dose diphenhydramine and/or hydrocortisone); so, there is no clinical trial experience to inform premedication strategies.

The authors note that lacking universal and standardized anti-AAV antibody titer assays hinders the ability to compare AAV gene therapy outcomes and products. We should emphasize that assay requirements and methods that are optimized for the clinical application of a specific AAV gene therapy product in a specific disease may differ from the requirements of an assay that might be developed to inform the understanding of AAV as a mature therapeutic platform for application across diseases. Miesbach and Srivastava reference the review by Gorovits et al. [7], which states, “As more experience is gained with regard to the predictability of clinical outcomes based on anti-AAV antibody status and titer, the standardization of antibody measurement would also enable a better comparison between clinical trials.” Data from the completed HOPE-B and the ongoing NCT06003387 [8] and NCT06008938 [9] trials are currently addressing the first portion of this statement, that is, gathering clinical experience regarding clinical outcomes based on anti-AAV antibody status.

At this time, a priority agreed upon by the sponsor of etranacogene dezaparvovec and the US Food and Drug Administration is NAb assay validation to enhance predictive value and safety monitoring for etranacogene dezaparvovec clinical application, with a focus on higher AAV5 titer ranges. Application of this newly validated assay in the ongoing NCT06003387 trial will extend the HOPE-B experience to provide additional clinical correlation of outcomes related to the specific AAV5 antibody assay [10] (in particular, relatively moderate and high NAb titers), for informed clinical decision-making at the individual patient level. A standardized, non–product-specific NAb assay, as suggested by the authors, could provide a different value by enabling crossproduct comparisons, helping to improve understanding of liver-directed systemic AAV gene therapy as a drug class. The clinical benefit of AAV gene therapy for hemophilia B, as summarized in the analysis by Miesbach and Srivastava, offers optimism that the opportunity for such comparisons may arise. The degree to which the lack of a non–product-specific AAV antibody assay represents a key limitation or a priority unmet need will depend on whether the pharmacoeconomic environment will support the clinical and commercial development of multiple AAV drugs for hemophilia.

We encourage the reader seeking additional individual participant-level data on AAV5 NAbs in HOPE-B to refer to the participant narratives in the Supplementary Materials of the studies by Raheja et al. [2] and Klamroth et al. [1] and the Supplementary Figures and narratives in Pipe et al. [6].
